# Supplementary material for: Iterative improvement in the automatic modular design of robot swarms
Source: PeerJ Comput Sci. 2020 Dec 7;6:e322. doi: 10.7717/peerj-cs.322 (PMC7924708; doi:10.7717/peerj-cs.322)
Supplement: Supplemental Information 3 [file peerj-cs-06-322-s003.zip › argos3/doc/api/standalone/a00406_source.html]

ARGoS: core/utility/string\_utilities.cpp Source File


- Main Page
- Related Pages
- Namespaces
- Classes
- Files

- File List
- File Members

# core/utility/string\_utilities.cpp

Go to the documentation of this file.

```
00001 
00010 #include "string_utilities.h"
00011 #include <cstdlib>
00012 #include <regex.h>
00013 
00014 namespace argos {
00015 
00016    void Tokenize(const std::string& str_string,
00017                  std::vector<std::string>& vec_tokens,
00018                  const std::string& str_delimiters) {
00019       // this taken from
00020       // http://oopweb.com/CPP/Documents/CPPHOWTO/Volume/C++Programming-HOWTO-7.html
00021 
00022       /* Skip delimiters at beginning */
00023       std::string::size_type lastPos =
00024                str_string.find_first_not_of(str_delimiters, 0);
00025 
00026       /* Find first "non-delimiter" */
00027       std::string::size_type pos = str_string.find_first_of(str_delimiters,
00028                                                             lastPos);
00029 
00030       while(std::string::npos != pos || std::string::npos != lastPos) {
00031          /* Found a token, add it to the vector */
00032          vec_tokens.push_back(str_string.substr(lastPos, pos - lastPos));
00033 
00034          /* Skip delimiters.  Note the "not_of" */
00035          lastPos = str_string.find_first_not_of(str_delimiters, pos);
00036 
00037          /* Find next "non-delimiter" */
00038          pos = str_string.find_first_of(str_delimiters, lastPos);
00039       }
00040    }
00041 
00042    /****************************************/
00043    /****************************************/
00044 
00045    std::string StringToUpperCase(const std::string& str_string) {
00046       char* buf = new char[str_string.length()];
00047       str_string.copy(buf, str_string.length());
00048 
00049       for(unsigned int i = 0; i < str_string.length(); ++i)
00050          buf[i] = toupper(buf[i]);
00051 
00052       std::string r(buf, str_string.length());
00053 
00054       delete[] buf;
00055 
00056       return r;
00057    }
00058 
00059    /****************************************/
00060    /****************************************/
00061 
00062    std::string StringToLowerCase(const std::string& str_string) {
00063       char* buf = new char[str_string.length()];
00064       str_string.copy(buf, str_string.length());
00065 
00066       for(unsigned int i = 0; i < str_string.length(); ++i)
00067          buf[i] = tolower(buf[i]);
00068 
00069       std::string r(buf, str_string.length());
00070 
00071       delete[] buf;
00072 
00073       return r;
00074    }
00075 
00076    /****************************************/
00077    /****************************************/
00078 
00079    void Replace(std::string& str_buffer,
00080                 const std::string& str_original,
00081                 const std::string& str_new) {
00082       /* Start from the beginning of the string */
00083       size_t unPos = 0;
00084       do {
00085          /* Look for the substring to replace */
00086          unPos = str_buffer.find(str_original, unPos);
00087          /* Has it been found? */
00088          if(unPos != std::string::npos) {
00089             /* Yes, it has been found */
00090             /* Replace the substring with the new one */
00091             str_buffer.replace(unPos, str_original.length(), str_new);
00092             /* Update unPos so that it point past the end of the replaced portion */
00093             unPos += str_new.length();
00094             /* Make sure unPos does not exceed the string length */
00095             if(unPos >= str_buffer.length()) {
00096                unPos = std::string::npos;
00097             }
00098          }
00099          /* Continue until the original string has been found */
00100       } while(unPos != std::string::npos);
00101    }
00102    
00103    /****************************************/
00104    /****************************************/
00105 
00106    bool MatchPattern(const std::string& str_input,
00107                      const std::string& str_pattern) {
00108       /* Code taken from
00109          http://www.opengroup.org/onlinepubs/000095399/functions/regexec.html
00110        */
00111       UInt32 nStatus;
00112       regex_t tRegExp;
00113       if(::regcomp(&tRegExp, str_pattern.c_str(), REG_EXTENDED | REG_NOSUB) != 0) {
00114          return false;
00115       }
00116       nStatus = ::regexec(&tRegExp, str_input.c_str(), 0, NULL, 0);
00117       ::regfree(&tRegExp);
00118       if (nStatus != 0) {
00119          return false;
00120       }
00121       return true;
00122    }
00123    
00124    /****************************************/
00125    /****************************************/
00126 
00127    std::string& ExpandEnvVariables(std::string& str_buffer) {
00128       size_t unStart = 0, unEnd;
00129       std::string strVarName;
00130       char* pchVarValue;
00131       bool bDone = false;
00132       do {
00133          /* Look for the $ character */
00134          unStart = str_buffer.find_first_of('$');
00135          /* Has it been found, and is it not the last character? */
00136          if(unStart != std::string::npos &&
00137             unStart+1 < str_buffer.length()) {
00138             /* Yes, it has been found */
00139             /* Look for the first non-alphanumeric character */
00140             unEnd = str_buffer.find_first_not_of("abcdefghijklmnopqrstuvwxyzABCDEFGHIJKLMNOPQRSTUVWXYZ0123456789_", unStart+1);
00141             /* Has it been found? */
00142             if(unEnd != std::string::npos) {
00143                /* Yes, it has been found */
00144                strVarName = str_buffer.substr(unStart+1, unEnd-unStart-1);
00145             }
00146             else {
00147                /* No, it has not been found */
00148                strVarName = str_buffer.substr(unStart+1, str_buffer.length()-unStart-1);
00149             }
00150             /* Get the variable value */
00151             pchVarValue = ::getenv(strVarName.c_str());
00152             /* Was the value found? */
00153             if(pchVarValue != NULL) {
00154                /* Yes, it was */
00155                /* Replace the variable name with its value */
00156                str_buffer.replace(unStart, strVarName.length()+1, pchVarValue);
00157             }
00158             else {
00159                /* Erase the variable name from the string */
00160                str_buffer.erase(unStart, strVarName.length()+1);
00161             }
00162             /* Reset the start pointer to prepare the next search */
00163             unStart = 0;
00164          }
00165          else {
00166             /* No further $ chars found or end of string reached */
00167             bDone = true;
00168          }
00169       } while(!bDone);
00170       return str_buffer;
00171    }
00172 
00173    /****************************************/
00174    /****************************************/
00175 
00176 }
```

---

Generated on 10 Jul 2018 for ARGoS by 
 1.6.1
